# Transcription factor ETV1 promotes angiogenesis after myocardial infarction via activation of the VEGFA/VEGFR2/eNOS pathway

**DOI:** 10.3389/fcvm.2025.1633438

**Published:** 2025-08-15

**Authors:** Jinyu Wang, Chunxia Li, Feng Li, Sen Fang, Yuan Chen

**Affiliations:** ^1^ Department of Geriatric Medicine, Shanxi Bethune Hospital, Shanxi Academy of Medical Sciences, Third Hospital of Shanxi Medical University, Tongji Shanxi Hospital, Taiyuan, China; ^2^ Third Hospital of Shanxi Medical University, Shanxi Bethune Hospital, Shanxi Academy of Medical Sciences, Tongji Shanxi Hospital, Taiyuan, China; ^3^ Departments of Geriatrics, Tongji Hospital, Tongji Medical College, Huazhong University of Science and Technology, Wuhan, Hubei, China

**Keywords:** myocardial infarction, ventricular remodeling, ETV1, angiogenesis, VEGF/VEGFR2/eNOS pathway

## Abstract

**Background:**

In our previous study, through integrative transcriptomic and ChIP-seq analysis, we revealed that ETV1 is a potential transcription factor involved in ventricular remodeling in the early stage of MI. This study aims to investigate the regulatory roles of ETV1 and whether ETV1 regulates angiogenesis after MI.

**Methods:**

In this study, MI model was induced by ligating the left anterior descending coronary artery. The expression of *Etv1* was modulated via intramyocardial injection of adeno-associated virus serotype 9 (AAV9) with endothelial-specific promoter *Icam2*. Fibrosis was determined by Masson staining and apoptosis was assessed by TUNEL staining. Angiogenesis was evaluated by CD31 immunofluorescence staining. For *in vitro* experiments, HUVECs were transfected with *ETV1* overexpression lentivirus, and wound healing and tube formation assays were performed to validate the angiogenic role of *ETV1*. Western blot was conducted to determine the level of angiogenetic factors and the underlying mechanisms.

**Results:**

The expression of *Etv1* was decreased in the hearts of MI mice, as well as in isolated cardiac microvascular endothelial cells (CMECs). Moreover, overexpression of *Etv1* alleviated the deterioration of heart function, mitigated the fibrosis, reduced apoptosis, and promoted angiogenesis after MI. Moreover, *ETV1* overexpression enhanced migration and tube formation abilities of HUVECs. Mechanistically, ETV1 upregulated the expression of VEGFA, VEGFR2, and eNOS.

**Conclusions:**

In summary, Etv1 promote angiogenesis via activating VEGFA/VEGFR2/eNOS pathway after MI, which further ameliorate adverse ventricular remodeling. These results suggest that ETV1 may serve as a potential target for the treatment of myocardial infarction.

## 1 Introduction

Acute myocardial infarction (AMI) is myocardial necrosis resulting from ischemia and hypoxia of the myocardium due to occlusion of coronary arteries (1). Numerous studies have shown that genes associated with inflammation, autophagy, apoptosis, and myocardial hypertrophy are activated after MI, ultimately leading to pathological ventricular remodeling (2, 3). Ventricular remodeling is the leading cause of arrhythmias, cardiac dysfunction, and heart failure after AMI (4).

There is evidence that early cardiac remodeling is partially reversible (4, 5). Angiogenesis, the sprouting of new capillaries from preexisting vessels, plays a vital role in promoting myocardial repair and alleviating adverse ventricular remodeling after MI. Das et al. demonstrated that the neonatal mouse heart can regenerate and repair itself through building collateral arteries in response to ischemic myocardial injury, but this capacity is lost in the adult mammalian heart due to impaired collateral artery formation (6). Meanwhile, neovascularization begins at the infarct border zone in the early stage of MI (7). However, due to inflammation and oxidative stress, angiogenesis is insufficient and unable to meet the metabolic demands of the ischemic myocardium, resulting in progression of pathological ventricular remodeling and aggravation of heart failure (8, 9). Therefore, exploring the regulatory mechanisms and therapeutic strategies for angiogenesis is of great significance.

By administering exogenous CXCL12, Das et al. reported that endothelial cells could be induced to migrate, proliferate, and reassemble into collateral arteries in the hearts of adult mice post-MI (6). In recent years, mesenchymal stromal cell (MSC)-based therapies and hydrogels-based therapies have gained much attention in promoting angiogenesis (10–12). Nevertheless, despite the progress that has been made, the poor biocompatibility and the potential cytotoxicity of these biomaterials remain to be solved.

ETV1 is a member of the ETS domain-containing transcription factor family. Emerging evidence indicates that ETV1 is implicated in the occurrence and metastasis of gastrointestinal cancer (13, 14). Sangphil et al. demonstrated that ETV1 facilitates colorectal tumorigenesis by binding to the FOXQ1 gene promoter (15). In addition, ETV1 is also found to be associated with prostate cancer progression (16, 17). Meanwhile, there are still relatively few studies exploring the roles of ETV1 in cardiovascular diseases. The current researches are mainly focused on atrial electrical and structural remodeling. Rommel et al. observed that cardiomyocyte-specific overexpression of ETV1 induces atrial arrhythmia, dilatation, and fibrosis in mice (18). Similarly, ETV1 is also reported to mediate the atrial remodeling induced by pressure overload (19). Our previous study deciphered that ETV1 is a potential transcription factor involved in ventricular remodeling in the early stage of MI (20). However, it is still unknown whether ETV1 regulates angiogenesis after MI.

In this study, we investigate the effect of ETV1 on angiogenesis and explore the potential mechanisms *in vitro* and *in vivo*. Our results indicate that ETV1 gene delivery improved cardiac function, reduced fibrosis, and increased angiogenesis. Consistently, *in vitro* overexpression of ETV1 promotes angiogenesis of HUVECs. Mechanistically, ETV1 exerts pro-angiogenic property through modulating VEGFA/VEGFR2/eNOS pathway.

## 2 Methods and materials

### 2.1 Animals

Male wild-type (WT) C57BL/6 mice (8weeks old) were purchased from Animal Center of Shanxi Medical University. All experiments were performed in accordance with the Guidelines for the Care and Use of Laboratory Animals published by the US National Institutes of Health, and the experiments were approved by the Animal Care and Use Committee of Shanxi Medical University.

### 2.2 Myocardial infarction

Mice were anaesthetized with chloral hydrate (300 mg/kg). Acute Myocardial infarction model was induced by ligation of left anterior descending coronary artery. The same procedure was performed in the sham group without LAD occlusion. Mice were sacrificed 1 week post-surgery for heart tissue collection.

### 2.3 AAV9 vectors construction and adult mice intracardiac injection

Etv1 overexpressing adeno-associated virus driven by endothelial specific gene Icam2 promoter (AAV2/9-Icam2-mEtv1-Flag-P2A-EGFP, AAV9-*Etv1*) or control viral vectors (AAV2/9-Icam*2*-EGFP, AAV9-NC) were constructed by Taitool Bioscience Co., Ltd (Shanghai, China) following standard methods (21). Briefly, the cDNA fragments encoding mouse ETV1 was cloned into inverted terminal repeat (ITR)-containing AAV9 plasmid harboring the human endothelial specific Icam2 promoter. AAV9 vectors, rep2/cap9 packaging plasmids, and helper plasmids were packaged in HEK293T cells (Thermo Scientific). After transfection using polyethylenimine for 72 h, cells were collected and lysed. AAV9 was purified and concentrated by gradient centrifugation. AAV9 titer was determined by qPCR. For the *in vivo* experiment, 3 days before MI surgery, mice received an intracardiac injection of AAV9-NC or AAV9-*Etv1* using an insulin syringe with a 30-gauge needle at a dose of 4 × 10^11^ viral genome particles per animal as previously described (22). Hearts were collected 1 week after MI.

### 2.4 Echocardiography

One week after MI surgery, mice were anaesthetized using 1.5%–2% isoflurane and placed in supine position. Heart function was assessed by echocardiography. Left ventricular ejection fraction (LVEF), left ventricular short-axis shortening rate (LVFS), left ventricular end-systolic diameter (LVIDs) and left ventricular end-diastolic diameter (LVIDd) were measured using the corresponding formulas.

### 2.5 Histology

Myocardial tissues from the infarct border zone were harvested at 7 days after MI surgery. Heart tissues were fixed in 4% paraformaldehyde and then dehydrated. After dehydration, the samples were embedded in paraffin and sectioned longitudinally. The sections were stored at −80℃ until further use.

### 2.6 Masson staining

Masson's trichrome staining was performed by using a kit (Solarbio) according to the manufacturer's protocol. The cardiac tissue sections from the infarct border zone were dewaxed and stained with Weigert's hematoxylin. After washing thoroughly with tap water and then rinsed with distilled water, the sections were stained with acid fuchsin solution, differentiated in phosphotungstic‒phosphomolybdic acid, stained with aniline blue, and washed with 1% acetic acid. After dehydration, the slides were mounted and scanned. The fibrotic area was measured with image J software.

### 2.7 TUNEL staining

Myocardial apoptosis in the infarct border zone was detected using a terminal deoxynucleotidyl transferase dUTP nick-end labeling (TUNEL) assay kit (Beyotime, China) according to the manufacturer's instructions. The slides were counterstained with DAPI for nuclei labeling. The fraction of apoptotic cells was estimated as ratio of TUNEL-positive cells to total cell nuclei.

### 2.8 Immunofluorescence

The sections from the infarct border zone were fixed with 4% paraformaldehyde. After permeabilization with 0.1% Triton X-100, the slides were blocked with 10% goat serum and then incubated with the primary antibodies against cTnT (Abcam, ab209813, 1:1,000) and CD31 (Abcam, ab222783, 1:1,000). The next day, the sections were washed and stained with the corresponding fluorescent secondary antibodies (Alexa Fluor-488 or Alexa Fluor-555, Abcam, 1:1,000). The nuclei were labeled with DAPI. Images were taken using an inverted fluorescence microscope (Olympus BX51, Japan). To assess the density of capillaries in border zones, the numbers of vessels were counted in 5 random fields on each section per animal and recorded as CD31^+^ vessels/mm^2^.

### 2.9 CMECs isolation

Cardiac microvascular endothelial cells (CMECs) were isolated as previously described (23). Briefly, the Mice hearts from each group were harvested and minced into small pieces. After digestion with collagenase type II and dispase, the cells were collected and incubated with CD31 magnetic beads (Miltenyi Biotec, Germany) for endothelial cell sorting. The isolated endothelial cells were cultured and used between passages 2 and 4 for subsequent western blot experiments.

### 2.10 Western blot analysis

Myocardial tissues from the infarct border zone and HUVECs were harvested and homogenized. The samples were lysed with RIPA buffer containing protease inhibitors. The concentration of protein was then quantified by BCA method. Equal amounts of total protein was separated by SDS-PAGE gels and then transferred to PVDF membranes. After blocking with 5% skim milk, the membranes were incubated with primary antibodies overnight against ETV1 (Abcam, ab314874, 1:1,000), VEGF Receptor 2 (CST, #2,479, 1:1,000), VEGFA (Abcam, ab214424, 1:1,000), eNOS (CST, #32,027, 1:1,000), and GAPDH (Abcam, ab9485, 1:1,000). The next day, the membranes were incubated with secondary antibodies, and protein signals were visualized using an Odyssey infrared imaging system. The gray value of protein bands was quantitatively analyzed by Image J software.

### 2.11 Cell culture

Human umbilical vein endothelial cells (HUVECs) were obtained from ATCC (American Type Culture Collection, USA). The cells were cultured in Endothelial Cell Growth Medium supplemented with 5% FBS, penicillin, and streptomycin. HUVECs between passages 3 and 5 were used for subsequent experiments.

### 2.12 Lentiviral vector construction and transfection

Lentivirus to overexpress ETV1 (Lenti-esEF1a-hETV1-Flag-IRES-MataGFP, Lenti-ETV1) and blank lentivirus (Lenti-esEF1A-3xFlag-IRES-MataGFP, Lenti-NC) were designed and constructed by Taitool Bioscience Co., Ltd (Shanghai, China). Briefly, human ETV1 cDNA was cloned into lentiviral vector at the MCS locus. The HEK293T cells were transfected with the lentiviral vector plasmid and packaging plasmids. The transfected cells were cultivated, and the supernatants were collected 72 h after transfection. Viral supernatants were concentrated using ultracentrifugation. Lentivirus titers were measured using FACS analysis and determined by infection of 293 T cells (24). Lentiviruses expressing GFP gene were used as the control. For the *in vitro* experiment, HUVECs were infected with lentiviruses at a multiplicity of infection of 3, and the transfection was performed according to the manufacturer's instructions. Briefly, cells were seeded at 1 × 10^5^/ml per well in 12-well plates. 2 × 10^8^ TU/ml *ETV1* overexpression lentivirus or 2 × 10^8^ TU/ml control lentivirus were added to HUVECs. The medium was changed 24 h post-transfection.

### 2.13 Scratch wound healing assay

Parallel lines were drawn on the back of 6-well plate. 5 × 10^5^ HUVECs were inoculated in 6-well plate and incubated overnight with 5% CO2 at 37℃. The next day, a scratch wound perpendicular to the parallel lines was made in the middle of the well using a 200 μl pipette tip. The cells were washed with PBS to remove the floating cells and then infected with lenti-*ETV1* or lenti-NC for 24 h. The area of scratch wound was photographed at 0 and 24 h following infection, and the distance was measured using ImageJ.

### 2.14 Tube formation assay

Before the experiment, Matrigel was thawed at 4°C overnight. Firstly, 50 μl Matrigel matrix was added to each well of a 96-well plate and incubated at 37°C for 30 min. After polymerization, 1 × 10^4^ HUVECs per well were seeded into the 96-well plate coated by Matrigel. Next, the cells were infected with lenti-*ETV1* or lenti-NC for 24 h. Tubule formation was then observed and photographed under an inverted microscope. The length of tubes and number of branch points were quantified using ImageJ software.

### 2.15 Statistical analysis

No statistical methods were used to predetermine sample sizes; however, our sample sizes were similar to those reported in a previous publication (25, 26). The results were presented as mean ± standard deviation (SD). Comparisons between groups were performed using Student's *t* test or one-way ANOVA followed by LSD *post hoc* test. *P* < 0.05 was considered to be statistically significant.

## 3 Results

### 3.1 Overexpression of ETV1 ameliorates cardiac dysfunction, alleviates apoptosis, and curtails fibrosis area of myocardial tissue after MI

**Figure 1:**
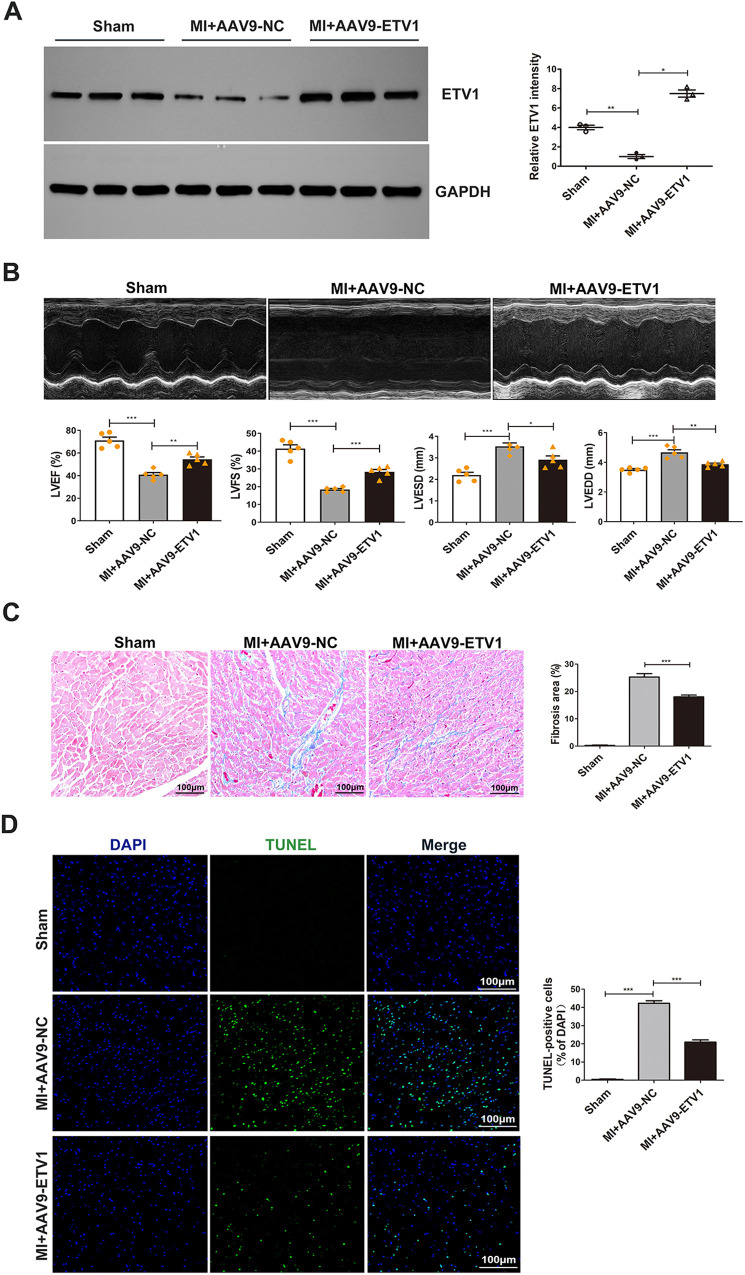
ETV1 overexpression attenuated cardiac dysfunction and pathological ventricular remodeling after MI. **(A)** The expression levels of *Etv1* in CMECs were examined by Western blot. GAPDH was used as loading control (*n* = 3). **(B)** Representative M-mode echocardiographic images at 7 days post-MI. Cardiac function was evaluated by echocardiography 7 days after surgery. Quantitative analysis was performed for left ventricular ejection fraction (LVEF), left ventricular fractional shortening (LVFS), left ventricular end-systolic diameter (LVEDs), Left Ventricular end-diastolic diameter (LVEDd) (*n* = 5). **(C)** Myocardial fibrosis was detected by Masson's trichrome staining. Blue represents fibrosis (*n* = 3). **(D)** Apoptosis was examined by TUNEL staining (*n* = 3). Scale bar = 100 *μ*m. Data were presented as mean ± SD. Comparisons among multiple groups were analyzed by one-way ANOVA followed by LSD *post hoc* test. *P < 0.05, **P < 0.01, ***P < 0.001, vs. indicated groups.

Our previous study demonstrated that ETV1 is a potential transcription factor involved in ventricular remodeling after AMI (20). To explore the role of ETV1 in the process of pathological ventricular remodeling after AMI, the expression level of *Etv1* in CMECs isolated from sham and MI mouse hearts were examined. Our data indicated that the ETV1 protein level was significantly downregulated in CMECs isolated from the infarct border zone compared with that in the sham group (Figure 1A). To further determine whether *Etv1* improves cardiac function after MI, *Etv1* was overexpressed in endothelium through intramyocardial injection of adeno-associated virus serotype 9 (AAV9) carrying the endothelial-specific promoter *Icam2* (AAV9- *Etv1*) 3 days before LAD ligation. Western blot showed that AAV9-*Etv1* injection resulted in significantly increased level of ETV1 in endothelial cells (Figure 1A). Cardiac functions were then monitored by echocardiography 1 week after MI surgery.

Our results showed that MI challenge significantly decreased left ventricular ejection fraction (LVEF) and left ventricular fractional shortening (LVFS), while left ventricular end-diastolic diameter (LVEDD) and left ventricular end-systolic diameter (LVESD) were significantly increased in AAV9-NC MI mice compared with sham mice. However, after transfection with AAV9-*Etv1*, the values for LVEF and LVFS were markedly higher, and the values for LVEDD and LVESD were markedly lower compared with AAV9-GFP mice (Figure 1B), indicating that *Etv1* ameliorates cardiac dysfunction after MI.

Myocardial fibrosis and apoptosis are important pathological mechanisms responsible for the development of pathological ventricular remodeling (PVR) and cardiac dysfunction after MI. We then investigated whether overexpression of *Etv1* could attenuate pathological ventricular remodeling. Masson staining showed that increased expression of ETV1 by AAV9-*Etv1* transfection significantly alleviated cardiac interstitial fibrosis compared with AAV9-NC MI mice in the border regions, as evidenced by reduced collagen deposition (Figure 1C). Moreover, *Etv1* overexpression significantly reduced cardiac apoptosis compared with the AAV9-NC MI mice (Figure 1D). These results suggest that ETV1 inhibited deterioration of cardiac function and at least partially ameliorated adverse cardiac remodeling in MI mice through its anti-fibrotic and anti-apoptotic effects.

### 3.2 Overexpression of ETV1 promotes angiogenesis after MI

**Figure 2:**
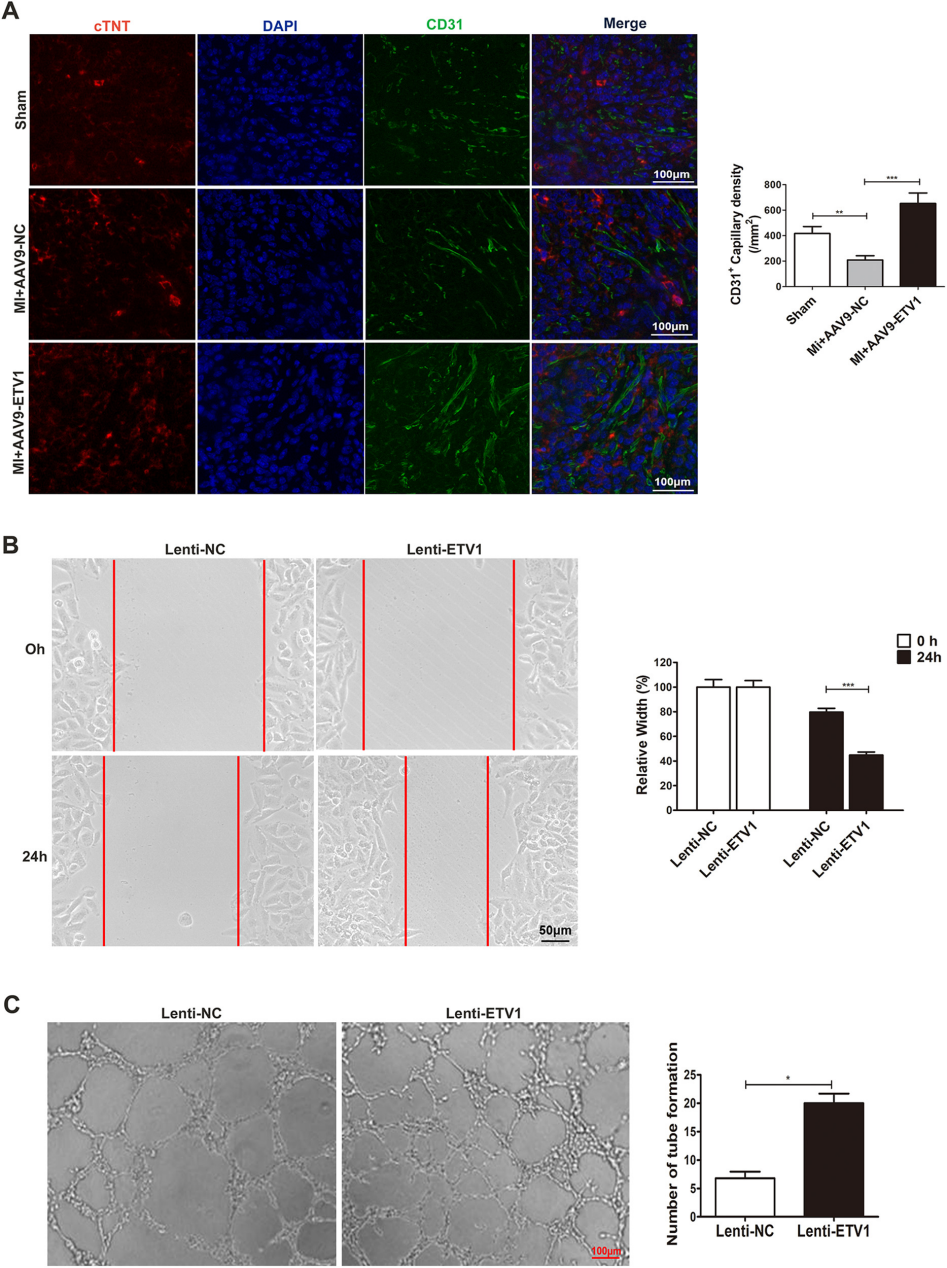
ETV1 overexpression promoted angiogenesis. **(A)** Immunofluorescence for cTNT, DAPI, and CD31 in border zone. Red represents cTNT labelling, and blue indicates nuclei stained with DAPI. Myocardial angiogenesis was examined by immunofluorescence staining for CD31 (green) (*N* = 3). Scale bar = 100 μm. **(B)** HUVECs were transfected with Lenti-ETV1 or Lenti-NC for 24 h. The migration ability of transfected HUVECs was measured by wound-healing assay (*n* = 3). Scale bar = 50 μm. **(C)** The angiogenic activity of transfected HUVECs was assessed by tube formation assay (*n* = 3). Scale bar = 100 μm. Data were presented as mean ± SD. Comparisons among multiple groups were analyzed by one-way ANOVA followed by LSD *post hoc* test. *P < 0.05, **P < 0.01, ***P < 0.001, vs. indicated groups.

Angiogenesis is essential for cardiac repair after MI. The insufficient neovascularization and subsequent shortage of oxygen supply in ischemic myocardium are major reasons leading to pathological ventricular remodeling and heart failure after MI. As mentioned previously, overexpression of *Etv1* alleviated adverse ventricular remodeling. To further explore the mechanisms of the protective role of *Etv1*, angiogenesis was assessed by CD31 immunofluorescence staining in the myocardium at 1 week after MI. Our results showed that overexpression of *Etv1* significantly increased the density of capillaries in the peri-infarct areas in comparison with AAV9-NC MI mice, as evidenced by increased CD31-positive vessels (Figure 2A), indicating that ETV1 enhances angiogenesis after MI.

### 3.3 Overexpression of ETV1 promotes migration and tube formation in human umbilical vein endothelial cells (HUVECs)

As mentioned above, overexpression of ETV1 promotes angiogenesis in mice after MI. In order to further clarify the effect of ETV1 on HUVECs cultured *in vitro* and validate the role of ETV1 in regulating angiogenesis, we transfected HUVECs with *ETV1* overexpression lentivirus (Lenti-*ETV1*) and negative control lentivirus (Lenti-NC) for 24 h under normoxia. The wound healing test and tube formation assay were then performed. The scratch experiment showed that overexpression of *ETV1* promoted the migration of HUVECs (Figure 2B). At the same time, transfection of HUVECs with Lenti-*ETV1* significantly increased the length and amount of tubes compared to Lenti-NC HUVECs (Figure 2C). Together, these results demonstrate that ETV1 played an important role in regulating cardiac angiogenesis.

### 3.4 Overexpression of ETV1 increases the expression of VEGFA, VEGFR2 and eNOS in the cardiac tissues after MI

**Figure 3:**
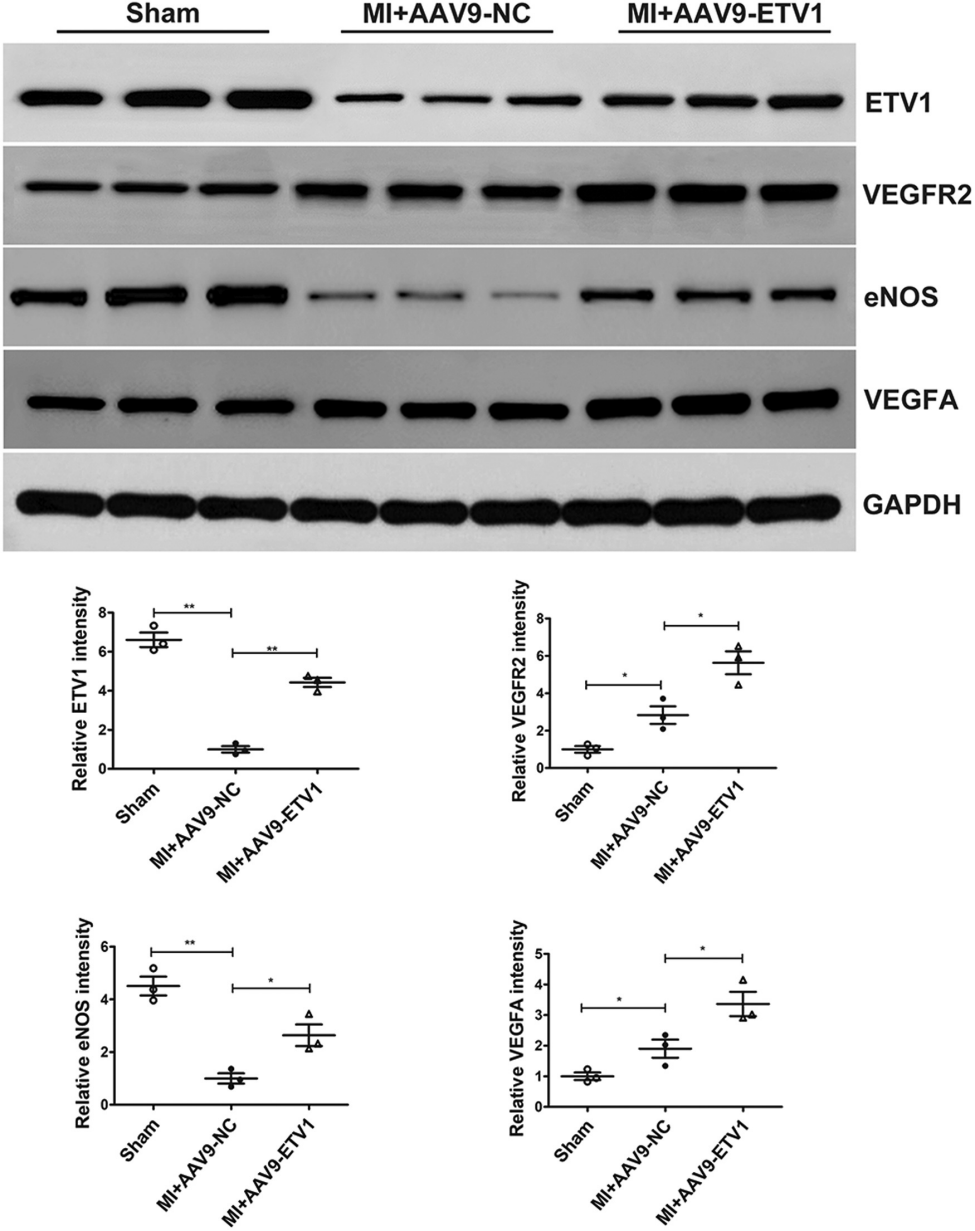
ETV1 overexpression increased the levels of VEGFA, VEGFR2 and eNOS in the cardiac tissues after MI. The protein levels of VEGFA, VEGFR2 and eNOS in the myocardium were examined by Western blot. GAPDH was used as loading control (*n* = 3). Data were presented as mean ± SD. Comparisons among multiple groups were analyzed by one-way ANOVA followed by LSD *post hoc* test. *P < 0.05, **P < 0.01, vs. indicated groups.

VEGF is a key angiogenetic factor and VEGF-VEGFR2 is considered as one of the most important pathways regulating angiogenesis. To gain insights into mechanisms underlying the proangiogenic role of ETV1, the levels of VEGFA and VEGFR2 were measured by western blot. Our data showed that the ETV1 protein level was significantly downregulated, and the levels of VEGF and VEGFR2 was significantly upregulated under MI conditions. More importantly, transfection with AAV9-*Etv1* further elevated the levels of VEGFA and VEGFR2 in comparison with AAV9-NC MI mice. eNOS is a critical modulator implicated in angiogenesis. Consistent with the expression of VEGFA and VEGFR2, overexpression of *Etv1* significantly increased the expression of eNOS (Figure 3). Collectively, our results suggest that ETV1 played an important role in upregulating the expression of pro-angiogenic factors, which further promoted endothelial cell proliferation, migration, and angiogenesis.

### 3.5 Overexpression of ETV1 increases the expression of VEGFA, VEGFR2 and eNOS in human umbilical vein endothelial cells (HUVECs)

**Figure 4:**
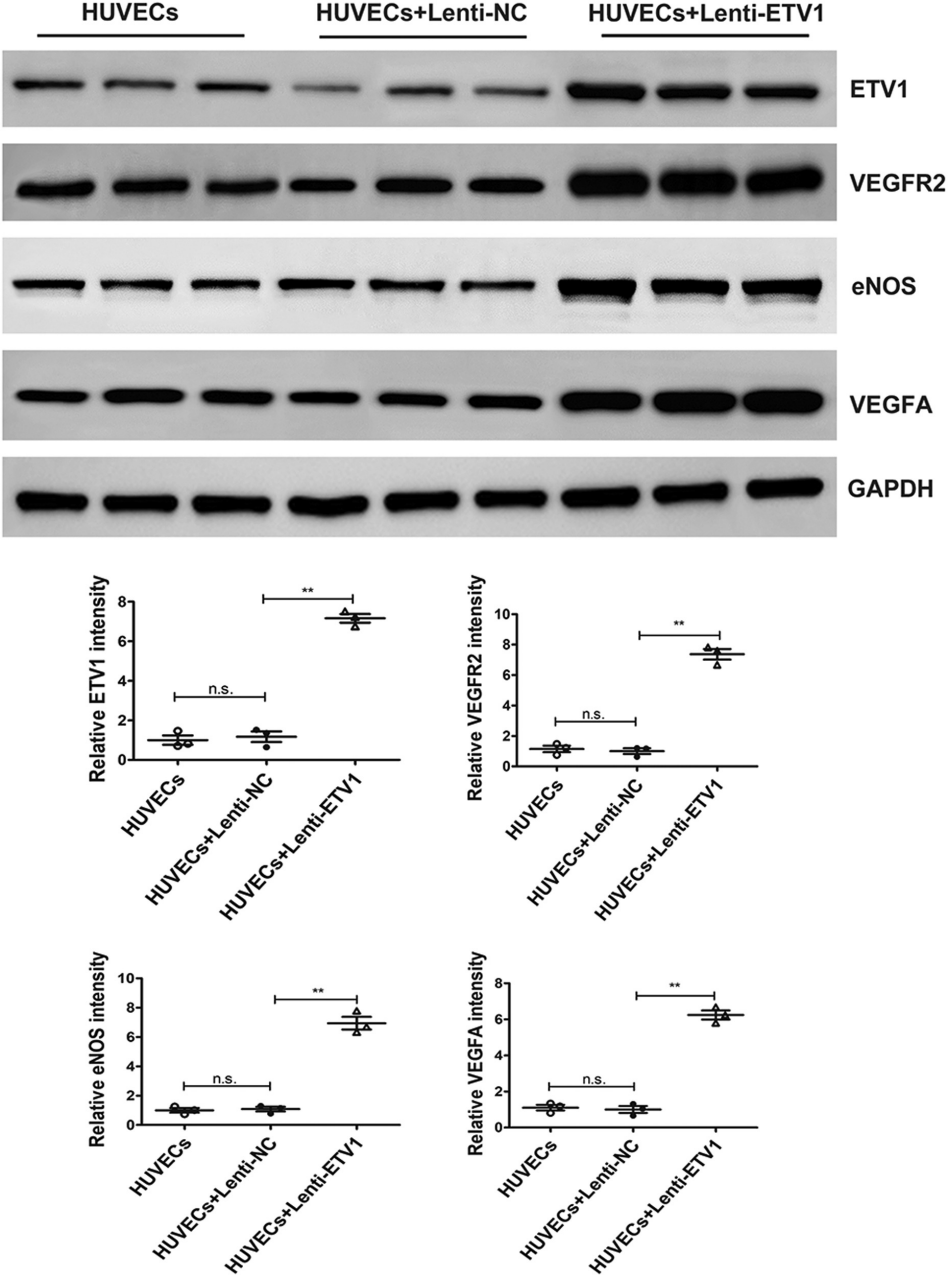
ETV1 overexpression increased the levels of VEGFA, VEGFR2 and eNOS in HUVECs. The protein levels of VEGFA, VEGFR2 and eNOS in transfected HUVECs were examined by Western blot. GAPDH was used as loading control (*n* = 3). Data were presented as mean ± SD. Comparisons among multiple groups were analyzed by one-way ANOVA followed by LSD *post hoc* test. *P < 0.05, **P < 0.01, n.s. indicates not significant, vs. indicated groups.

Our *in vitro* experiment demonstrated that overexpression of ETV1 promoted endothelial migration and tube formation in HUVECs. As described above, overexpression of ETV1 increased the levels of angiogenetic factors (eNOS, VEGFR2, VEGFA) after MI. To investigate the molecular mechanisms of proangiogenic effects of ETV1 on HUVECs, we then also examined the effect of ETV1 on expression of eNOS, VEGFR2, and VEGFA in HUVECs after transfection with Lenti-*ETV1*. Consistent with the expression pattern *in vivo*, transfection of HUVECs with Lenti-*ETV1* significantly elevated the levels of VEGF, VEGFR2, and eNOS compared with Lenti-NC HUVECs (Figure 4). Taken together, these data demonstrate that ETV1 promoted angiogenesis via VEGF/VEGFR2/eNOS signaling pathway.

## 4 Discussion

Our previous study demonstrated that ETV1 is a potential transcription factor involved in regulating gene expression and ventricular remodeling after AMI; In this study, we found that the expression of ETV1 was downregulated after MI, and endothelial-specific overexpression of ETV1 mitigated the deterioration of cardiac function, inhibited myocardial apoptosis and fibrosis, and promoted angiogenesis after MI; *in vitro* experiments showed that overexpression of ETV1 promotes the migration and tube formation of HUVECs. These alterations were associated with the activation of VEGF/VEGFR2/eNOS.

Percutaneous coronary intervention is an effective method to re-establish blood flow and protect ischemic cardiomyocytes after AMI (27). However, in-stent restenosis still remains a serious clinical problem. Moreover, the long-term application of antiplatelet drugs also increases the risk of postoperative bleeding, especially gastrointestinal bleeding in elderly patients (28). On the other hand, current drugs including calcium channel inhibitors and nitrate ester improve blood supply mainly by dilating the coronary artery. At present, there are no drugs targeting angiogenesis approved for the treatment of ischemic cardiomyopathy.

Angiogenesis is defined as the sprouting of capillaries from pre-existing vessels (29, 30). Early neovascularization is of great significance in reducing the necrotic area, attenuating cardiac dysfunction, and inhibiting pathological ventricular remodeling (31); However, unlike excessive abnormal tumor angiogenesis, angiogenesis in the infarct border zone is limited and restricted as a result of multiple factors such as persistent local inflammation and oxidative stress (8, 9, 32–34). Meanwhile, due to the relatively insufficient angiogenesis, the ischemic myocardium is unable to obtain adequate supply of oxygen and nutrients, resulting in further ischemic damage and necrosis (35). Recently, therapeutic angiogenesis has gained much attention in the treatment of ischemic heart disease (36). Some natural active molecules, mesenchymal stromal cell (MSC)-based therapies, and hydrogels-based therapies have been proven to promote angiogenesis in animal experiments (37, 38).

ETV1 is a member of the ETS family of transcription factors. Accumulating evidence has highlighted the crucial role of ETV1 in gastrointestinal stromal tumors. The effect of ETV1 on atrial remodeling has also been revealed. However, whether ETV1 can modulate angiogenesis after MI remains to be uncovered. In this study, endothelial-specific overexpression of ETV1 improved cardiac dysfunction, inhibited apoptosis and fibrosis post-MI, indicating attenuation of pathological cardiac remodeling. Meanwhile, angiogenesis is often measured by determining CD31-positive vessel density (39). Our *in vivo* study showed that ETV1 overexpression significantly increased the density of CD31-positive capillaries. The proliferation and migration of endothelial cells are prerequisites for angiogenesis (31). To investigate the impact of ETV1 on endothelial cells, HUVECs were transfected with ETV1 overexpression lentivirus, and subsequent experiment indicated that ETV1 enhanced the migration and tube formation of HUVECs. These results suggested that ETV1 inhibited pathological myocardial remodeling by promoting angiogenesis.

Mechanically, we further observed that the expression of VEGF, VEGFR2, and eNOS was significantly upregulated after overexpression of ETV1. Meanwhile, unlike the expression pattern in CMECs, our results showed that the expression of ETV1 in the hearts of AAV9-ETV1 MI mice was lower than that in the sham mice. This difference can be explained by the fact that, apart from endothelial cells, cardiomyocytes and fibroblasts constitute the predominant cell types in the heart (40, 41). VEGF/VEGFR2 is considered as one of the most important pathways responsible for angiogenesis (42). Previous studies have demonstrated that VEGF promotes the proliferation and migration of endothelial cells by binding to its receptor, VEGFR, thus facilitating angiogenesis (43). As a member of the VEGF family, VEGFA plays a major role in angiogenesis (44). However, the generation of endogenous VEGFA is relatively inadequate after MI and unable to induce sufficient angiogenesis for the repair of injured myocardium (45). Our study found that VEGFA expression increased after MI. Moreover, overexpression of ETV1 further elevated the expression of VEGFA, which contributed to the enhanced angiogenesis. As a receptor for VEGF, VEGFR2 is the main mediator of VEGFA-induced angiogenesis (46). Consistent with the expression pattern of VEGFA, the expression of VEGFR2 was also up-regulated after ETV1 overexpression. The above results suggested that ETV1 promotes angiogenesis by regulating the VEGF/VEGFR2 signaling pathway.

eNOS is a downstream effector of the VEGF/VEGFR2 signaling pathway and mediates VEGF-induced angiogenesis by catalysing the production of NO (47). Studies have proved that NO promotes the migration and proliferation of endothelial cells, thereby modulating angiogenesis after MI (48, 49). Our study exposed that eNOS was upregulated after overexpression of ETV1, which resulted in enhanced angiogenic capabilities of endothelial cells.

In summary, our study revealed that ETV1 promotes angiogenesis after MI via the VEGFA/VEGFR2/eNOS pathway. However, this study also has limitations. Firstly, this study did not decipher the mechanisms of ETV1 in regulating angiogenesis at transcriptional level, whether ETV1 could directly regulate the expression of VEGFA expression need further exploration. Secondly, we did not perform ETV1 knockdown experiments to further validate the regulation of ETV1 on angiogenesis. Thirdly, our *in vivo* experiment demonstrated that ETV1 inhibited myocardial apoptosis. However, whether ETV1 suppresses endothelial cell apoptosis requires further investigation. Furthermore, our study mainly focused on the effects of endothelial ETV1 overexpression on angiogenesis and ventricular remodeling. However, given the well-documented high affinity of AAV9 for cardiomyocytes, we cannot exclude the possibility that ETV1 could also be overexpressed in cardiomyocytes and the cardioprotective effects of ETV1 may be partially attributed to its direct regulatory role in cardiomyocytes. Despite these shortcomings, our study suggested a potential role of ETV1 in therapeutic angiogenesis for ischemic heart disease.

## Data Availability

The original contributions presented in the study are included in the article/Supplementary Material, further inquiries can be directed to the corresponding author.
